# Portal of medical data models: information infrastructure for medical research and healthcare

**DOI:** 10.1093/database/bav121

**Published:** 2016-02-11

**Authors:** Martin Dugas, Philipp Neuhaus, Alexandra Meidt, Justin Doods, Michael Storck, Philipp Bruland, Julian Varghese

**Affiliations:** 1 Institute of Medical Informatics, University of Münster, Germany; 2 European Research Center for Information Systems (ERCIS)

## Abstract

**Introduction**: Information systems are a key success factor for medical research and healthcare. Currently, most of these systems apply heterogeneous and proprietary data models, which impede data exchange and integrated data analysis for scientific purposes. Due to the complexity of medical terminology, the overall number of medical data models is very high. At present, the vast majority of these models are not available to the scientific community. The objective of the Portal of Medical Data Models (MDM, https://medical-data-models.org) is to foster sharing of medical data models.

**Methods**: MDM is a registered European information infrastructure. It provides a multilingual platform for exchange and discussion of data models in medicine, both for medical research and healthcare. The system is developed in collaboration with the University Library of Münster to ensure sustainability. A web front-end enables users to search, view, download and discuss data models. Eleven different export formats are available (ODM, PDF, CDA, CSV, MACRO-XML, REDCap, SQL, SPSS, ADL, R, XLSX). MDM contents were analysed with descriptive statistics.

**Results**: MDM contains 4387 current versions of data models (in total 10 963 versions). 2475 of these models belong to oncology trials. The most common keyword (*n* = 3826) is ‘Clinical Trial’; most frequent diseases are breast cancer, leukemia, lung and colorectal neoplasms. Most common languages of data elements are English (*n* = 328 557) and German (*n* = 68 738).

Semantic annotations (UMLS codes) are available for 108 412 data items, 2453 item groups and 35 361 code list items. Overall 335 087 UMLS codes are assigned with 21 847 unique codes. Few UMLS codes are used several thousand times, but there is a long tail of rarely used codes in the frequency distribution.

**Discussion**: Expected benefits of the MDM portal are improved and accelerated design of medical data models by sharing best practice, more standardised data models with semantic annotation and better information exchange between information systems, in particular Electronic Data Capture (EDC) and Electronic Health Records (EHR) systems. Contents of the MDM portal need to be further expanded to reach broad coverage of all relevant medical domains.

**Database URL**: https://medical-data-models.org

## Introduction

Medical data models describe data structures of information systems in medicine. For example, a medical history form of a clinical trial contains data elements regarding previous diseases like myocardial infarction. This list of data elements—including properties like element name, element description and data type—can be considered a data model. These models are of key importance to build study databases, because they determine what kind of data analysis is possible for any medical topic of interest. Despite many initiatives for transparency in clinical research [such as AllTrials (1)], most medical data models are not available to the scientific community, neither in medical research nor in routine healthcare.

The search space for medical data models has astronomical dimensions: A typical documentation form consists of approximately 40 data elements. The Systematized Nomenclature of Medicine Clinical Terms (SNOMED CT) (2) contains >300 000 non-synonymous concepts, i.e. there are at least 300 000 options for a data element. This corresponds to $\left(\begin{array}{c} 300.000\\ 40 \end{array}\right)\;\approx \;\text{1,5E171}$ possible documentation forms, many more than atoms in the universe (∼1E80).

The subset of medically useful data models is certainly much smaller, but still very large: In the field of medical research, approximately 200 000 clinical studies are registered (3). The average amount of case report forms (CRFs) per patient in a clinical trial increased from 55 to 180 pages in recent years (4). Therefore >10 million different CRFs were used in these clinical studies. Because of this variability and complexity, information systems in medicine constitute a big data challenge. Eligibility criteria are available on the Internet, but cover only 1–2 pages out of approximately 100 pages per trial, therefore the vast majority of those forms is not directly available to the scientific community.

In routine healthcare a disease-specific data model is needed to address all relevant patient attributes. The current international classification of disease [ICD version 10 (5)] lists >13 000 diagnoses. Approximately 400 data elements (6) are needed per diagnosis in routine healthcare, corresponding to more than 5 million data elements. However, data models in routine healthcare are not yet standardised and multilingual—on a global basis patients report their symptoms in 200+ languages –, therefore much more than 5 million data elements are actually being used. Regarding routine healthcare most data models are not available to the public, because they are implemented within commercial software products.

Because medical data models are not accessible to the scientific community, re-use of data models is very limited and ‘the wheel is re-invented’ worldwide in medical information systems.

The objective of the Portal of Medical Data Models (MDM) (7) is to overcome this lack of transparency. MDM is a registered German and European information infrastructure (8, 9), i.e. it provides shared and sustainable access to scientific services. Specifically, it provides a multilingual platform for exchange and discussion of data models in medicine, both for medical research and healthcare. In the following, a short overview of the technical approach is given and a detailed analysis of currently available contents for the scientific community is provided.

## Methods

### IT architecture and software tools

**Figure 1:**
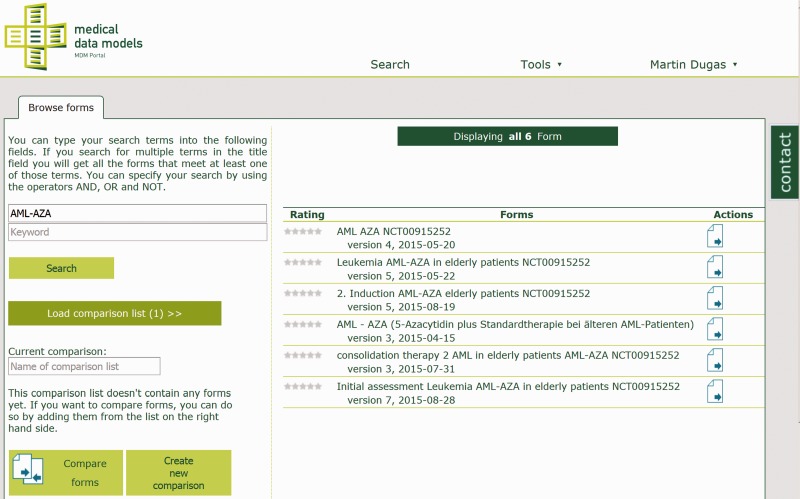
Search for clinical trial ‘AML-AZA’ on the MDM portal, resulting in six data models.

**Figure 2:**
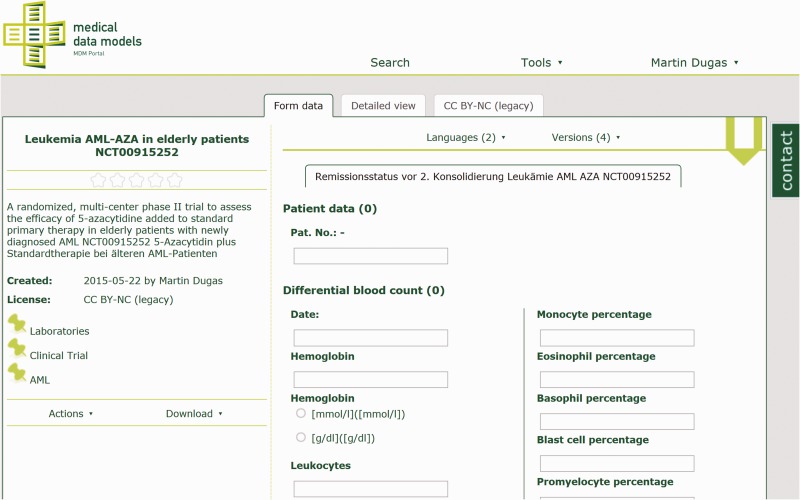
Laboratory data model from AML-AZA trial with hemoglobin, leukocytes and other parameters. Semantic codes and complete code lists for each data item are available in the detailed view.

The technical approach of the MDM portal has been described previously (10). In summary, medical data models are stored in CDISC ODM (11) format on a web server. ODM structures are parsed and transferred to a MySQL database. Converters for several export formats of data models (12, 13) are integrated into the portal (see Table 1). Semantic annotation with Unified Medical Language System (UMLS) codes (14, 15) is provided for the majority of data elements. Software components of the portal are written in Java, Ruby on Rails and R. Registered users can search (Figure 1), view (Figure 2), download and comment data models. Dedicated users can upload new data models with version control. A web-based editor for data models is integrated into the portal.

**Table 1.** Export formats of the MDM portal

ODM	CDISC operational data model
PDF	Portable document format
CDA	HL7 clinical document architecture
CSV	comma separated values
MACRO-XML	Format for EDC system MACRO
REDCap	Format for EDC system REDCap
SQL	Database template
SPSS	IBM SPSS Syntax file
ADL	Archetype description language (ADL)
R	R statistics language
XLSX	Microsoft Excel format

### Analysis of portal contents

The MDM portal database was analysed using R scripts (16) with the library RMySQL. The time course of available data models was analysed, i.e. the cumulative number of data models from the start of the system until 2015. In CDISC ODM data items are structured by item groups which are organised in forms. Each data item is characterized by a name, e.g. ‘patient age’, a data type, such as ‘integer’, and optional translations as well as one ore more UMLS codes. Each data model can be updated (via upload or integrated editor), for instance by creation of a new version. Only the latest version of a data model was counted to determine the total number of models. The time course of created and updated data models was analysed. Number of versions per data model was described with a frequency distribution.

Most frequent keywords and their combinations were analysed with an UpSet plot (17). Keywords are based upon medical subject headings (MeSH) (18) with custom extensions. Data models were categorised into the following domains: clinical trial, electronic health record (EHR), registry, quality assurance and other (e.g. can be used in more than one domain). Most frequent data model types were determined in general and specifically for clinical trial-related forms.

UMLS codes are used for semantic annotations in the MDM portal. Descriptive statistics for semantic annotation were generated: (i) Number of semantically annotated data items, itemgroups and code lists; (ii) number of unique UMLS codes; (iii) overall frequency distribution of UMLS codes and number of UMLS codes per data item; (iv) number of UMLS coded items per data model. MDM is a multilingual system, therefore most frequently used languages of data items were determined.

## Results

### Data models

**Figure 3:**
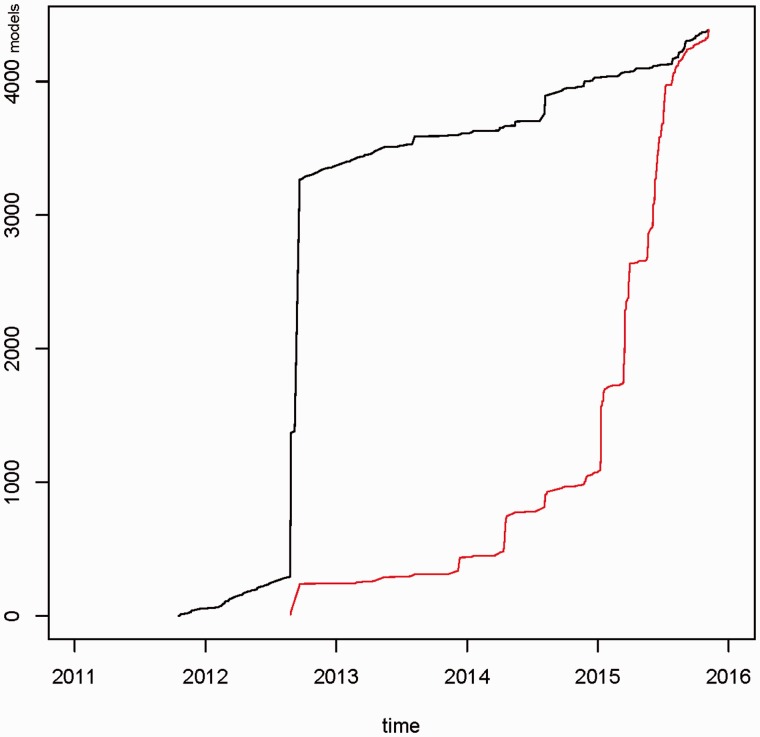
Cumulative number of newly created data models (black graph) and updated data models (red graph) for the time period 2011–2015. In 2012, a draft set of ∼3000 models was uploaded into the portal. In 2015 ∼75% of data models were updated. In total 4387 data models were available.

Figure 3 presents the total number of data models between 2011 and 2015. In the third quarter of 2012 a large set of models was uploaded. These models were available from Internet sources and were processed using custom-built converters. In the first quarter of 2015 a large proportion of these models was updated, e.g. typing errors were corrected and UMLS codes were modified. In total, 4387 data models were available (as of November 2015). In a period of three months (August–October 2015) 78 266 data models were viewed by portal users and 354 models were downloaded.

**Figure 4:**
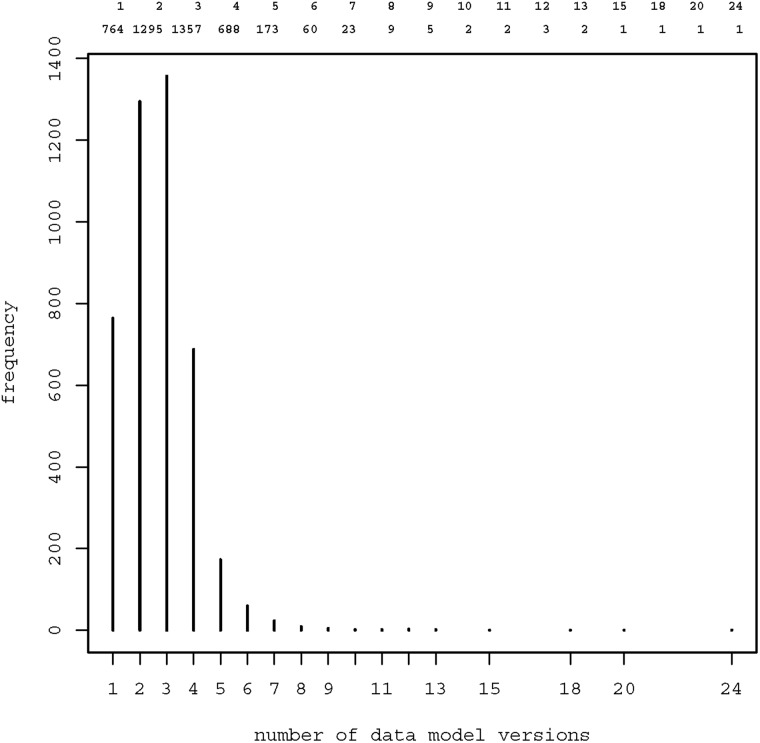
Frequency distribution of data model versions. Most models were available in two (*n*  =  1295) or three (*n*  =  1357) versions. 13 models were provided in 10 or more versions.

Figure 4 shows the frequency distribution of data model versions: Overall, there were 10 963 model versions, median 3 per model (range 1–24). These model versions contained 62 327 item groups, 397 403 items and 111 891 code lists. Most frequent data types of items were text (55.6%), boolean (14.2%), date (10.4%), integer (10.0%) and float (9.4%).

### Keywords

**Figure 5:**
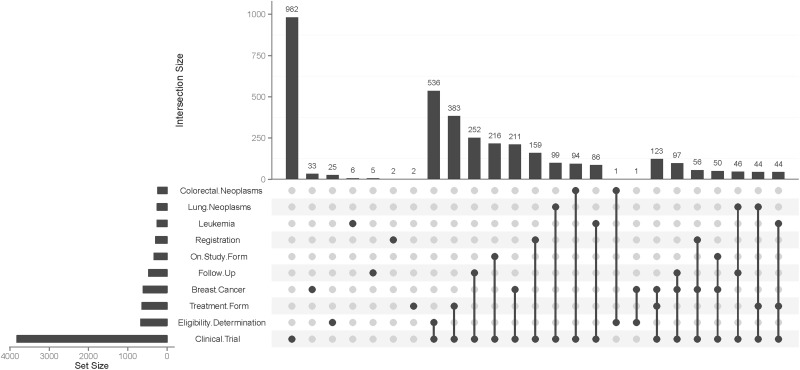
UpSet plot of 10 most frequent keywords. The bar chart on the left indicates the frequency of keywords: ‘Clinical Trial’ is the most common keyword (almost 4000 occurences). The upper bar chart indicates the intersection size of keyword combinations. ‘Clinical Trial’ and ‘Eligibility Determination’ is the most frequent combination of keywords. The most common triple is ‘Clinical Trial’ – ‘Treatment Form’ – ‘Breast Cancer’.

Each data model can be tagged with one or several keywords from the MeSH thesaurus. Figure 5 presents most frequent keywords and their combinations as an UpSet Plot. Clearly, most contents of the MDM portal were derived from clinical trials. Most frequent diseases were breast cancer, leukemia, lung and colorectal neoplasms. Because eligibility forms of clinical trials are available on the Internet, ‘Eligibility Determination’ is a frequent keyword. Table 2 presents the number of data models by major disease area. The majority of data models belonged to oncology. In addition, there were disease-independent models, e.g. regarding discharge letters.

**Table 2:** . Frequency of data models by major disease area

Major disease area	#Data models
Oncology	3109
Inflammatory or infectious disease	81
Neuroscience	98
Cardiovascular	112
Diabetes	46
Respiratory	11

201 data models were derived from EHR systems. Top EHR model types were patient discharge, medical history taking and clinical conference. Most frequent disease-specific EHR models were related to prostatic neoplasms, breast cancer and leukemia. 114 models were derived from registries, predominantly from oncological and neurological registries. Quality assurance was addressed in 71 models, mainly derived from German AQUA forms (19). These forms cover all domains of mandatory quality assurance in Germany[(>4 million documented cases (20)]. In addition, there were 176 models which can be used both in a clinical and a research setting.

### Semantic annotation

Regarding current model versions, semantic annotations were available for 108 412 items, 2453 item groups and 35 361 code list items. Overall 335 087 UMLS codes were assigned with 21 847 unique codes. Most frequent medical concepts were Laboratory Procedures (C0022885) and Physical Examination (C0031809).

**Figure 6:**
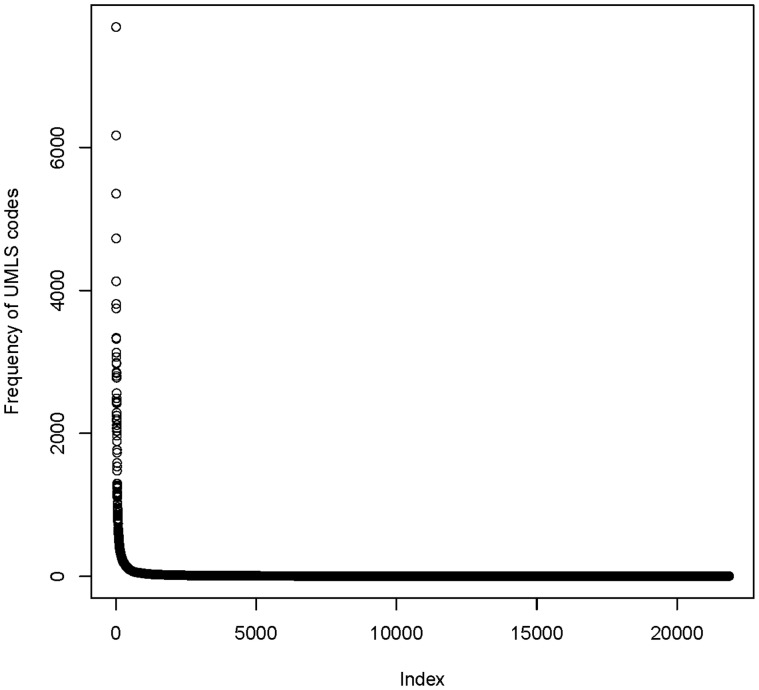
Frequency distribution of 21 847 unique UMLS codes in the MDM portal. Few codes are used very often (>1000 fold), but there is a long tail of rarely used codes.

Figure 6 shows the frequency distribution of UMLS codes. The median number of occurrences per UMLS code was only 1, with a wide range (1–7,685). This is an indicator for the semantic richness of medical data items: there is a long list of UMLS codes which was used infrequently.

**Figure 7:**
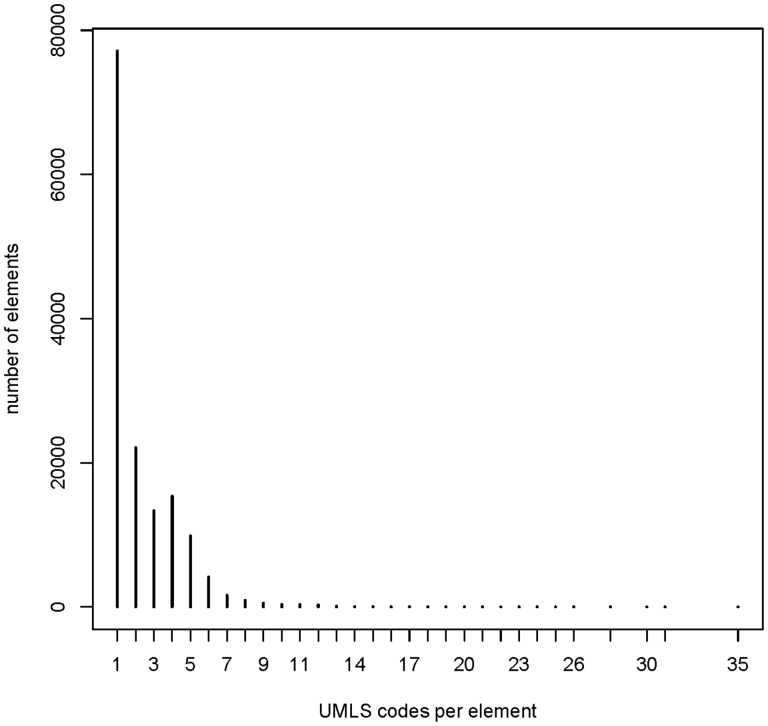
Frequency of UMLS codes per annotated element: median 1 (range 1–35). Overall 146 226 annotated elements (108 412 items, 2453 item groups and 35 361 code list items).

The frequency of UMLS codes per annotated element (items, item groups and code list items) is presented in Figure 7. The median number of codes per element was 1, with maximum of 35. This indicates that there are few elements with a high number of UMLS codes, for example complex eligibility criteria.

The median number of UMLS coded items per data model was 20 (range 0–478). Most common languages of elements were English (*n* = 328 557) and German (*n* = 68 738). In multilingual forms, there were text elements for each covered language.

## Discussion

At present, most medical data models are not available to the scientific community, but there are important advantages of model sharing and Open Metadata (21). Compatible data structures are of key importance for data exchange and integration in medicine. Medical data models should be harmonised as much as possible to enable data integration and analysis for research purposes and to avoid duplicate data entry in healthcare. As outlined in the introduction, there are a huge number of medical data models. Therefore an information infrastructure is needed to support sharing and discussion of data models in medicine.

The portal of medical data models started with approximately 250 models in 2012 (9). As of November 2015 it contains more than 4300 models, in most cases derived from clinical trials. In general, a large proportion of data models is related to oncology. More than 330 000 UMLS codes are assigned to data items, item groups and code lists. UMLS codes were chosen because they provide the largest coverage of medical concepts. Most codes are assigned by human experts. A small set of semantic codes is used very often, but the frequency distribution has a very long tail: i.e. there are many different UMLS codes which are used only once. 4300 models is a considerable number, but there are >13 000 diagnoses in the international classification of diseases (ICD-10 (5))—and each diagnosis will probably have disease-specific data elements: The ICD-10 disease category, e.g. diabetes mellitus type I (E10) is too granular. For each diabetes complication—such as coma (E10.0) or eye complications (E10.3) —additional data items are required. This indicates that much more data models are needed to provide a broad coverage of all medical domains.

In general, copyright laws regarding data models need to be respected. In our experience the copyright status of many data models is not clearly specified. This impedes re-use of models in research and routine care. From our perspective more widespread use of standardised licenses like creative commons (22) would be very helpful to foster sharing of data models.

Several Electronic data capture (EDC) systems started to provide CRF libraries to facilitate re-use of data collection instruments. For instance, REDCap (23) provides such a CRF library. It is a popular EDC system from Vanderbilt University with >1500 institutional partners worldwide. The REDCap library started with 128 instruments (24) and now expanded to 930 data collection forms (as of September, 2015). Since REDCap version 6.5.0 (released May 2015) the MDM portal is a directly linked external instrument library of REDCap. The PhenX toolkit (25), funded by the U.S. National Human Genome Research Institute, is another external REDCap library. This toolkit contains 485 forms in REDCap format (as of September, 2015). OpenClinica is another well-known open source EDC system in clinical research, which provides a CRF library (26) (20 CRFs, as of September, 2015). The cancer Data Standards Registry and Repository (caDSR) from NCI (27) provides a form builder with 4033 released forms and a total amount of 50 553 common data elements (as of September, 2015), available in Excel format and also via REST-interface. Common data elements are also defined in the NINDS-project with >10 000 items and 1000 CRF modules (28). The Clinical Information Modeling Initiative (29) contains approximately 400 item groups (as of September, 2015).

Data models are also being published in the field of EHR systems: Clinical Document Architecture (CDA) from HL7 (30) is currently the most established industry standard in EHR systems. The implementation guide regarding CDA for clinical notes (31) lists 27 document level templates, 71 Section level-templates and 109 entry-level templates (as of June, 2015). OpenEHR Clinical Knowledge Manager (32) provides 15 EHR-related templates and 407 archetypes (as of June, 2015). There are several initiatives that do not manage forms but rather specify and discuss data elements: The Clinical Element Model contains >5000 concepts (as of July, 2015) (33). The United States Health Information Knowledgebase comprises 12 forms and approximately 200 data elements with semantic annotations (34). Metadata Online Registry of the Australian Institute of Health and Welfare contains >2000 released data elements (as of July, 2015) (35).

This list of data model resources regarding EDC and EHR systems is not complete. But almost each system is using its own technical format for data structures (REDCap-, OpenClinica-, caDSR-, CIMI-, HL7-CDA-, openEHR-format). The MDM portal intends to foster data model sharing between systems with different technical formats: Each data model can be exported in several formats (see Table 1). The MDM portal applies CDISC ODM (11), which is an open standard, supported by regulatory authorities: CDISC ODM/Define XML is part of FDA’s Data Standards Catalog, which was announced to become mandatory for new drug applications by end of 2016 (36). The MDM portal leverages several data model converters from ODM to other data structures.

Another important feature of the portal is semantic annotation. Based on UMLS coding, data elements are semantically enriched to avoid ambiguities due to synonyms and homonyms within the biomedical domain. Semantic codes enable comparative analysis of data models: For instance, what data elements are identical or similar between two data models? (37). Potential key data elements for specific medical domains can be identified by systematic analysis of most frequent concept codes (6, 38). >335 000 codes are already assigned to items, item groups and code list values in the MDM portal. Certainly manual curation and validation of these codes is needed. Semi-automatic methods, i.e. expert-based semantic annotation with computer-based suggestions, will stay important in the future (despite fully-automated approaches) (39). However, semantic annotation will be even more complicated for weakly structured, non-standardized and probabilistic data sets in personalised medicine (40). At this stage it became evident that few codes like ‘Date in time’ are used very often, but there is a long tail of rarely used semantic codes.

### Future work

Given the semantic complexity of medicine, much more data models need to be processed to reach a broad coverage. It is planned to deliver another 20 000 data models in the next three years with guidance from an external advisory board of domain experts. A close collaboration with the University Library of Münster was established to make the MDM portal sustainable—both from a technical and a contents perspective. Regular user surveys are planned to guide further development accordingly. A single institution is certainly not capable to provide all relevant content; therefore the MDM portal applies a community-based approach. We encourage medical researchers worldwide to contribute their data models and use the MDM portal as a platform for collaboration.
